# Observation and Prevention of Caregiver-Related Medication Errors in Community Pharmacies: A Cross-Sectional Study Across Urban, Town, and Rural Settings

**DOI:** 10.3390/ph19081148

**Published:** 2026-07-24

**Authors:** Rafael Toledo, Pilar Zafrilla, Begoña Cerdá, Mercedes Guilabert, José Joaquín Mira, Amparo Talens, Pura Ballester-Navarro

**Affiliations:** 1 Faculty of Pharmacy and Nutrition, UCAM Universidad Católica de Murcia, 30107 Murcia, Spain; rtoledo3@alu.ucam.edu (R.T.); mpzafrilla@ucam.edu (P.Z.); bcerda@ucam.edu (B.C.); 2 Programa Internacional de Doctorado en Ciencias de la Salud, UCAM Universidad Católica de Murcia, 30107 Murcia, Spain; 3 Health Psychology Department, Miguel Hernandez University, 03202 Elche, Spain; mguilabert@umh.es (M.G.); jose.mira@umh.es (J.J.M.); 4 Fundación para el Fomento de la Investigación Sanitaria y Biomédica de la Comunitat Valenciana, 03010 Alicante, Spain; 5 Centro de Salud Hospital-Plá, 03013 Alicante, Spain; 6 Servicio Farmacia Hospitalaria, Departamento de Salud de Alicante, Hospital General Universitario Dr. Balmis, 03010 Alicante, Spain; talens_amp@gva.es

**Keywords:** medication errors, caregivers, older adults, community pharmacy, rural health, patient safety, polypharmacy

## Abstract

**Background**: Medication errors committed by informal caregivers of older adults represent a significant risk to patient safety, particularly in the context of population aging and increasing therapeutic complexity. Community pharmacies could play a key role in detecting and preventing them; furthermore, pharmacy location (urban, town, or rural settings) role in medication errors remains underexplored. **Objective**: To analyze the types and frequency of caregiver-related medication errors observed by community pharmacists and to determine if differences exist according to pharmacy location. **Methods**: A cross-sectional study was conducted using an online self-reported questionnaire by pharmacists, technicians, and assistants working in the Region of Murcia, Spain. The questionnaire assessed types of medication errors observed, factors associated, preventive strategies, professional attitudes, and strategies at home. Descriptive statistics were calculated, and associations between pharmacy location and categorical variables were evaluated using Pearson’s chi-square (χ^2^) test or Fisher–Freeman–Halton exact tests. Statistical significance was set at *p* < 0.05. **Results**: A total of 148 professionals participated, including 109 pharmacists (73.6%), 24 pharmacy technicians (16.2%) and 15 pharmacy assistants (10.1%). According to pharmacy location, 74 participants worked in urban pharmacies (50.0%), 63 in town pharmacies (42.6%) and 11 in rural pharmacies (7.4%). The most frequently reported medication errors were incorrect administration time (n = 91, 61.5%), omission errors (n = 87, 58.8%) and dosing errors. (n = 80, 54.1%). Treatment complexity and polypharmacy were identified as the main contributing factors. Most preventive strategies and professional practices showed no statistically significant differences detected for most preventive strategies and reported professional practices across settings. However, moderate associations were found regarding the perceived frequency of medication and prescribing errors in rural settings reporting lower frequencies. However, findings involving the rural subgroup should be interpreted cautiously due to a limited sample size. **Conclusions**: Community pharmacy professionals demonstrate a consistent and proactive role in detecting and preventing caregiver-related medication errors across urban, town, and rural settings. Although geographic location has limited influence on most professional practices, rural pharmacies may assume an expanded role in patient and caregiver support. Standardized prevention strategies could be implemented broadly, with additional adaptations for rural contexts.

## 1. Introduction

In recent decades, population ageing has increased exponentially, representing one of the major challenges for healthcare systems worldwide [1]. This phenomenon is mainly due to increased life expectancy and declining birth rates. According to the World Health Organization, it is estimated that by 2030, one in six people in the world will be over 60 years of age, and that by 2050, the number of individuals in this age group will double compared with current levels [1]. This demographic change is closely associated with an increased prevalence of chronic diseases and polymedicated patients, a minimum of five simultaneous ongoing medications, leading to a greater complexity in the management and monitoring of our elders, their healthcare, therapeutic regimens and pharmacological treatments [1,2].

In Spain, the number of polymedicated patients has increased significantly over the last decade, contributing to a rise in healthcare demand and healthcare expenditure [3,4]. Several studies have shown that polypharmacy is associated with an increased risk of drug interactions, adverse events, hospitalizations, and functional decline in older individuals [5,6,7]. Furthermore, the complexity of therapeutic regimens has been linked to lower treatment adherence and a higher risk of experiencing adverse events [8].

Medication-related errors currently represent one of the leading causes of preventable harm in healthcare systems worldwide [5,7,9,10]. In older adults, these errors may have particularly significant consequences, as they happen in a structure with age-related physiological changes that compromise medication metabolism or elimination [9]. Among the most common medication errors described in the literature are dose omissions, duplications after omissions, incorrect administration, or confusion associated with naming or packaging [3,11,12]. These errors may compromise treatment effectiveness and increase the risk of medication-related adverse events.

In Spain, medicines are primarily prescribed by physicians, dentists and podiatrists within their legally recognised areas of competence, while nurses may indicate, use and authorize the dispensing of certain medicines and health products within the limits established by national regulation. Community pharmacists do not diagnose or replace the prescriber; however, Spanish legislation defines community pharmacies as healthcare establishments responsible for the acquisition, custody, conservation and dispensing of medicines, and recognises pharmacists’ role in providing information and contributing to the rational use and follow-up of pharmacological treatments [13,14,15]. Having that in mind and considering that many medication errors occur in the home setting, where patients must independently manage increasingly complex treatments. Several studies suggest that factors such as low health literacy, difficulties in understanding medical instructions, or insufficient communication with healthcare professionals may increase the risk of medication-related errors. Furthermore, the limited duration of medical consultations, which in many cases has been substantially reduced, together with the high workload in primary care, may hinder patients from receiving the necessary information to ensure the appropriate use of their treatments.

In this context, community pharmacies play a crucial role in patient education regarding the safe use of medicines. As patients regularly collect their prescribed medicines from community pharmacies, dispensing encounters provide repeated opportunities to clarify treatment instructions, resolve medication-related concerns and reinforce the correct use of medicines. These interactions can be adapted to the individual needs of patients and are not necessarily restricted to a predefined consultation time. Patient counselling, medication review and the identification or prevention of medication problems are integral components of pharmaceutical care [16]. As pharmacies represent easily accessible points within the healthcare system, community pharmacists are in a strategic position to identify medication-related problems, detect potential errors, and provide health education to patients to prevent them [17].

Interventions such as structured medication review, medication reconciliation, and pharmacotherapeutic follow-up have proven effective in improving treatment safety and reducing adverse events associated with polypharmacy [18,19]. In fact, several studies have indicated that medication reviews conducted by community pharmacists through targeted questioning prior to dispensing may reduce emergency department visits and improve clinical outcomes in polymedicated older patients [20]. In addition to these professional interventions, patients often use various strategies to avoid medication errors, such as the use of pill organisers, electronic reminders, mobile applications, or personalised dosage systems (PDS) [20,21,22] However, despite these tools, it is estimated that a significant proportion of medication-related errors could be prevented through improved health education and increased monitoring by healthcare professionals [21].

Furthermore, it should be noted that many older adults present some degree of functional dependency and require the assistance of caregivers for the correct administration of their treatments [6,23,24]. These caregivers, who are often relatives or individuals without specific healthcare training, play a key role in medication management at home [23]. The administration of complex treatments, the coordination of multiple medications, and the interpretation of medical instructions may represent difficult tasks for caregivers, thereby increasing the risk of medication administration errors [25]. Among the most common caregiver-related errors are incorrect administration schedules, dose omissions, dosing errors, or the use of non-prescribed medications [26]. Several studies have highlighted that family caregivers often require greater support and specific tools from healthcare professionals to efficiently manage medications for older patients [27]. Therefore, community pharmacies may represent an essential point of contact for caregivers, as pharmacists are among the most accessible healthcare professionals and can provide education and guidance regarding the correct use of medications and any related concerns [28].

Despite the growing evidence regarding the role of community pharmacists in the prevention of medication errors [17,20,28,29], there is currently limited research specifically focused on errors committed by caregivers and on how these are identified and addressed in daily community pharmacy practice. Moreover, the geographical context may influence pharmaceutical professional practice, access to healthcare services, and the relationship between patients and healthcare professionals. In rural settings or small municipalities, where access to other healthcare services is often limited, pharmacists tend to assume a broader role in the health education of patients and caregivers [7].

For these reasons, the present study aims to analyze medication-related errors committed by caregivers of polymedicated older adults from the perspective of community pharmacists. In addition, the study seeks to identify which errors professionals consider the most prevalent and relevant, as well as the strategies that can be implemented within community pharmacies to prevent or correct them. Finally, the study will evaluate whether differences exist in the detection, correction, and prevention of these errors according to the location of the pharmacy, urban, town, or rural, to contribute to the development of context-adapted prevention strategies aimed at improving medication safety among older adults.

## 2. Results

The final sample consisted of 148 participants, of whom 101 were women (68.2%), representing an approximate 2:1 female-to-male ratio. The predominant age range in the overall sample, as well as in urban and town settings, was 40–59 years; however, row-level analyses using exact tests where appropriate indicated that the 60–80-year category was proportionally more frequent in rural pharmacies (*p* = 0.0099; η^2^ = 0.0759), whereas the 40–59-year category did not reach statistical significance after the revised analysis (*p* = 0.0990).

The participants represented different geographic areas: 18% were in urban transit areas; 12% in urban residential or neighborhood areas; 20% in urban neighborhoods with a high proportion of older residents; 22% on the main street of a town; 21% in town neighborhood areas; and 7% in rural areas.

Significant differences were observed in sex distribution of professionals by pharmacy location (*p* = 0.0020; η^2^ = 0.0857), with a higher proportion of women in most urban and town settings and a higher relative proportion of male professionals in rural areas. Regarding professional category, 109 participants were pharmacists (73.6%), 24 were pharmacy technicians (16.2%), and 15 were pharmacy assistants (10.1%). Most professional variables showed broadly comparable distributions across settings. Years of professional experience, professional category, and pharmacy location showed no statistically significant differences after the revised row-level analysis, with the pharmacy counter remaining the predominant setting for error detection and prevention.

As shown in Table 1, the sociodemographic and professional characteristics of participants were broadly comparable across pharmacy settings, although some specific row-level differences were observed for the 60–80 year age category (*p* = 0.0099), sex distribution (*p* = 0.0020), and more than 30 years of professional experience (*p* = 0.0355). Pharmacy professionals aged 60–80-years, male professionals and those with more than 30 years of professional experience were proportionally more frequent in rural pharmacies, whereas female professionals were proportionally less frequent. No statistically significant differences were observed in the remaining categories related to age, professional experience, professional role or setting of pharmaceutical care. Findings regarding rural settings should be interpreted cautiously due to the small number of respondents.

### 2.1. Types of Errors and Associated Factors

The distribution of medication errors reported by community pharmacy professionals was broadly comparable across urban, town and rural settings, indicating a consistent pattern despite pharmacy location, except for incorrect medication preparation, which showed a statistically significant association with pharmacy setting and was more frequently reported in urban pharmacies. The most frequently identified errors were incorrect administration timing (61.5%), omitted doses (58.8%), and dosing errors (54.1%). Similarly, treatment complexity (83.8%) emerged as the factor most associated with these errors across all settings.

Potential triggers for medication errors followed a broadly comparable pattern across the different settings, with most comparisons remaining non-significant after the use of exact tests where required. Analgesics showed a non-significant trend towards being more frequently associated with medication errors than other therapeutic groups (*p* = 0.0729).

As shown in Table 2, although the overall distribution of medication errors was similar across settings, certain specific patterns can be observed. Errors related to incorrect medication preparation showed a statistically significant association with pharmacy location after exact testing where appropriate (*p* = 0.0190), suggesting that this type of error may be influenced by contextual or organizational factors. Additionally, while most causes and contributing factors were consistently reported across all settings, communication-related issues and caregiver-related factors remained prominent, highlighting the complexity of medication management beyond purely pharmacological aspects. Differences were also observed in the types of dosage forms and therapeutic groups involved in errors, although these variations did not reach statistical significance.

### 2.2. Error Prevention Strategies

Strategies implemented to prevent medication errors were generally similar by location. The most common preventive actions were asking open-ended questions, reviewing medication before dispensing, and providing ongoing education to patients or caregivers, with most comparisons remaining non-significant after the use of exact tests where expected counts were low.

When witnessing an error, 94.59% of respondents reported correcting it directly and clearly whenever detected, with no relevant differences by location.

As shown in Table 3, pharmacy professionals demonstrated a consistent and structured approach to detecting and managing medication errors across all settings. In addition to commonly used detection strategies, such as open-ended questioning and medication review, the data highlight the importance of tailored communication depending on the clinical condition, particularly in chronic and complex diseases. Diabetes (*p* = 0.0068) and neurological or psychiatric diseases (*p* = 0.0275) showed statistically significant associations with pharmacy location after exact testing, suggesting that patient education needs may vary depending on the setting. Furthermore, the findings reflect a strong emphasis on continuous interaction with caregivers, both for ensuring understanding and for preventing future errors.

### 2.3. Professional Communication with Patients

A large majority of participants reported using clear and concise language, paying careful attention to the way they communicate and dedicating a considerable amount of time to patient communication regardless of their current workload (>90%), with no statistically significant differences observed according to pharmacy location. Furthermore, no major variations were detected in the perceived preparedness to correct medication errors, level of professional involvement, empathy, or self-assessment of communication style after the revised row-level analysis (Table 4).

As shown in Table 4, pharmacy professionals reported a high level of professional engagement in the management of medication errors, regardless of pharmacy location. Most respondents indicated that they adopt a direct and clear approach when addressing errors, reflecting a proactive and intervention-oriented practice. In addition, the perception of professional responsibility in error prevention was consistently high, with most participants recognising this role as an integral part of their practice. Despite minor variations in responses, no statistically significant differences were observed between settings, suggesting broadly similar reported professional approaches across geographical contexts.

Nevertheless, significant associations were found between sex and pharmacy location, with a predominance of female professionals in urban areas and towns, whereas in rural areas the proportion was reversed, with 8 men compared to 3 women (*p* = 0.0020; η^2^ = 0.0857). The effect size indicates an association of small to moderate magnitude.

Regarding the frequency with which medication errors committed by caregivers of older adults are detected, variability was observed depending on the location of the pharmacy. The effect size (η^2^ = 0.124) suggests an association of moderate magnitude.

Concerning the frequency with which prescribing errors are detected, professionals working in rural areas most frequently selected the option “rarely”, whereas in urban areas and towns, the most selected response was “occasionally”. The option “frequently” was rarely selected across all locations. The effect size (η^2^ = 0.176) indicates a moderate-to-high association, suggesting that pharmacy location has a more marked influence on this variable.

The four questions referred to as “case scenarios” were also answered by all participants. Case scenario 1 was correctly answered by 88 respondents, representing 59.46% of the total sample. Case scenario 2 was correctly answered by 85.81% of the healthcare professionals, whereas the correct response rates for case scenarios 3 and 4 were 75.67% and 38.51%, respectively.

As shown in Table 5, rural community pharmacy professionals described a multifaceted role in supporting medication management and caregiver needs. More than half of the respondents reported occasionally undertaking additional educational or follow-up activities (54.5%), while most perceived education and emotional support as part of their professional role at least occasionally (81.8%). Geographical barriers were also considered relevant to caregiver-related medication errors, with 72.7% of the respondents indicating that these barriers contributed “a lot” or “quite a lot”. Among the specific challenges identified, lack of health training was the most frequently reported (63.6%), followed by lack of family coordination (45.5%). These findings provide a descriptive overview of the perceived educational and support needs in rural practice; however, they should be interpreted cautiously because of the small number of rural respondents and the absence of a direct comparison group in Table 5.

Because Table 5 includes only professionals working in rural settings, these findings are descriptive and should not be interpreted as comparative results between pharmacy locations.

## 3. Discussion

The present study analyses the observation and actions of community pharmacy professionals in response to medication errors committed by caregivers of older adults, determining whether differences exist according to the location of the pharmacy. The findings suggest a notable degree of uniformity across the different settings, although this interpretation should be considered in light of the cross-sectional design, the self-reported nature of the data, and the small rural subgroup.

Similarities in Reported Professional Practices Across Settings

These similarities are observed both in sociodemographic variables and in aspects related to error detection, intervention strategies, communication with patients, and perceptions of professional roles. These results suggest that professional practice in community pharmacy maintains a relatively uniform foundation regardless of the geographical environment in which it is carried out.

These findings are consistent with the existing literature on community pharmacy practice, which describes that pharmacists perform similar roles in health education, detection of medication-related problems, and patient counselling [30]. Furthermore, several studies have highlighted that the involvement of community pharmacists in medication safety represents a key element in the care of older patients, particularly in reducing adverse effects among polymedicated individuals and those with chronic conditions [31].

In our study, participants demonstrated a proactive attitude towards the observation and correction of medication errors, reporting that they correct errors directly whenever they detect any anomaly. In addition, respondents agreed that community pharmacies should be actively involved in the prevention of medication errors. This perspective is consistent with previous research emphasizing the importance of medication review interventions in older adults to optimize medication use and improve the safety of pharmacological treatments [32].

Most Common Errors and Associated Factors

Regarding the types of medication errors reported by community pharmacy professionals, incorrect administration timing (n = 91, 61.5%), omitted doses (n = 87, 58.8%) and dosing errors (n = 80, 54.1%) were the most frequently identified. These findings are consistent with previous evidence showing that medication management at home requires caregivers to perform multiple organizational and cognitive tasks, including interpreting treatment instructions, coordinating administration schedules, preparing doses and adapting to treatment changes. The predominance of timing, omission and dosing errors may therefore be related to the difficulty of coordinating multiple medicines, dosing intervals, meals and daily routines, particularly when therapeutic regimens are complex. Although the present study did not directly assess clinical consequences, these errors may compromise therapeutic effectiveness or increase the risk of adverse drug events. Medication regimen complexity has been associated with non-adherence, medication self-administration errors, caregiver-related administration difficulties, and, in some studies, higher hospitalization rates. Therefore, the high reported frequency of these errors should be regarded as a clinically relevant signal supporting the need for systematic caregiver education and periodic review of medication management routines [33,34].

With respect to the factors contributing to the occurrence of errors, treatment complexity was the most frequently reported factor (n = 124, 83.8%), followed by communication or comprehension difficulties and the high number of prescribed medicines. Medication regimen complexity extends beyond the number of medicines, and may also involve different dosage forms, dosing frequencies, administration instructions and the need to coordinate treatment with meals or daily routines. These characteristics may increase the organizational and cognitive demands placed on caregivers, who may be required to prepare doses, coordinate multiple administration schedules, supervise medication intake, and adapt medication management following treatment changes. Previous evidence has associated greater medication regimen complexity with poorer medication adherence and in some studies, higher rates of hospitalisation among older adults. Therefore, the prominence of treatment complexity in the present study may help to explain the high frequency of administration-time, omission, and dosing errors reported by pharmacy professionals. However, because these relationships were not directly assessed, this interpretation should be considered explanatory rather than causal [35,36].

From a clinical perspective, dosing errors deserve particular attention because administration of an excessive dose may increase the risk of toxicity and adverse drug events, whereas an insufficient dose may reduce therapeutic effectiveness. Omitted doses may similarly compromise treatment control, particularly in chronic diseases requiring sustained pharmacological management. Errors in administration time may also be clinically relevant when the effectiveness or safety of a medicine depends on maintaining specific dosing intervals or coordinating administration with meals. Although the present study did not directly assess patient outcomes, medication errors in home care should not be considered merely procedural events, as they may contribute to preventable medication-related harm. This concern is particularly relevant in older adults receiving multiple medicines, who may be more vulnerable to the consequences of medication-related problems. Previous reviews have identified caregiver medication administration errors at home as a potentially important patient-safety issue and have highlighted the need for caregiver training and tailored support strategies [33,37].

The statistically significant association observed for incorrect medication preparation deserves particular attention. This type of error was reported by 49 participants (33.1%) and was more frequently identified in urban pharmacies (43.2%) than in town (25.4%) or rural settings (9.1%; *p* = 0.0190). Medication preparation in the home may require caregivers to identify the correct medicine, organise doses and administration schedules, update medication aids following treatment changes and translate written instructions into daily medication routines. These tasks may become more demanding when several medicines or complex regimens are involved and when caregivers have limited training or insufficient professional support. Previous studies have highlighted the complexity of medication management by informal caregivers [38,39] and the importance of caregiver education and tailored support to reduce medication-related risks in the home setting [26,33]. Nevertheless, the present study did not assess the specific mechanisms underlying incorrect medication preparation or measure workload, dispensing volume, or the complexity of individual prescriptions. Therefore, the higher proportion reported in urban pharmacies should not be attributed to specific contextual or organisational factors on the basis of the present data alone. Rather, this finding should be considered exploratory and supports reinforcing clear and practical counselling on medication preparation and verifying caregiver understanding during dispensing encounters.

The significant associations observed for diabetes (*p* = 0.0068) and neurological or psychiatric diseases (*p* = 0.0275) also warrant consideration. Diabetes management may involve the coordination of medication administration with meals, glucose monitoring, recognition and prevention of hypoglycaemia, and, in some cases, the correct use of injectable medicines or devices [40]. These tasks may become particularly demanding in older adults with functional or cognitive limitations and may increase their reliance on caregivers. Current recommendations therefore emphasise the need to individualise treatment, simplify medication regimens when appropriate, and involve the patient’s support network in diabetes management [40]. Neurological and psychiatric diseases comprise a heterogeneous group, and the present questionnaire did not collect diagnosis-specific information; therefore, the mechanisms underlying this association cannot be determined. Nevertheless, evidence from people living with dementia, an important subgroup within neurological disorders, shows that cognitive decline and complex medication regimens may increase difficulties in medication management and create a greater need for clear, practical, and tailored information for both patients and caregivers [41,42]. Such information should address medication indications, expected effects, safe administration, and treatment changes [41]. These findings may support condition-specific counselling in community pharmacies, including simple verbal explanations, written instructions, verification of caregiver understanding, and coordination with other healthcare professionals. However, given the small rural subgroup and the number of comparisons performed, these associations should be interpreted as exploratory rather than as evidence of definitive geographical differences.

Significant Differences According to Location

Most variables showed no statistically significant differences across pharmacy settings; however, some specific associations were observed. First, differences were identified in the distribution by sex according to location (*p* = 0.0020), with a relatively higher proportion of male professionals in rural areas. However, the effect size was small to moderate, indicating that this difference, although statistically significant, is not structurally determinant.

Second, the perceived frequency of caregiver-related medication errors and prescribing errors varied across pharmacy settings, with rural respondents more frequently selecting the category “rarely”, particularly for prescribing errors. However, these findings should not be interpreted as evidence that medication errors are objectively less frequent in rural areas, because the study assessed professionals’ self-reported perceptions rather than the actual incidence of errors. Furthermore, only 11 professionals worked in rural pharmacies; therefore, the stability and generalisability of these findings are limited.

The rural-specific findings presented in Table 5 should be interpreted descriptively rather than as evidence of statistically significant differences between pharmacy settings. Within the rural subgroup, 6 of 11 respondents (54.5%) reported occasionally undertaking additional educational or follow-up functions, while 7 of 11 (63.6%) perceived geographical barriers as contributing substantially to caregiver-related medication errors. Lack of health training (63.6%) and lack of family coordination (45.5%) were the most frequently reported barriers. These findings may indicate a perceived need for greater educational support and care coordination in rural practice, but they do not demonstrate that rural pharmacy professionals assume a broader role than professionals working in urban or town settings.

Previous research conducted in rural and remote communities has suggested that limited access to other healthcare services may increase interest in broader community pharmacy services and reinforce the potential contribution of pharmacists to local healthcare provision [43]. Nevertheless, differences between healthcare systems and the small rural sample in the present study require cautious interpretation. Further studies with larger and more balanced samples are needed to determine whether healthcare accessibility, continuity of care, or caregiver-support needs contribute to geographical differences in medication-safety practices.

Professional Role and Communication

Another relevant finding was the high level of professional responsibility, empathy, and communication competence reported by participants. Most respondents indicated that they corrected caregiver-related medication errors immediately (n = 134, 90.5%), considered communication style highly relevant to treatment adherence (n = 131, 88.5%), and described themselves as prepared or very prepared to correct errors without generating guilt (n = 141, 95.3%). These findings suggest a patient-centred approach in which clear, empathetic, and non-judgemental communication is considered an essential component of medication-safety practice. However, because these data were self-reported, the high proportion of favourable responses may partly reflect social desirability and should not be interpreted as direct evidence of observed professional behaviour.

The communication strategies reported in this study also provide relevant practical information. Plain language was widely used, and information repetition and written instructions were frequently selected as methods for supporting caregiver understanding. These strategies may help reduce the cognitive burden associated with complex medication regimens, particularly when caregivers must remember several medicines, doses, schedules, or treatment changes. Nevertheless, repeating information or asking whether it has been understood may not always be sufficient to identify misunderstandings. Family caregivers may experience difficulties obtaining clear medication information, deciding which healthcare professional to consult, and interpreting therapeutic instructions, highlighting the need for communication that is adapted to their health-literacy level and medication-management responsibilities [37,44].

In this context, active verification of understanding may complement verbal explanations and written information. The teach-back method involves asking the recipient to explain, in their own words, how the medication should be used, allowing the healthcare professional to identify and correct misunderstandings. Evidence from a systematic review suggests that teach-back can improve knowledge, information recall, self-care abilities, and medication-related behaviours across different healthcare settings, although the included studies were heterogeneous and long-term evidence remains limited [45]. In community pharmacy practice, this approach could be adapted to caregivers by asking them to describe how they will prepare and administer the medication at home. This may be particularly useful after treatment changes or when regimens involve multiple medicines, complex schedules or special administration instructions.

Furthermore, caregivers should be recognized as essential partners in health outcomes, and it is therefore necessary to strengthen collaboration between caregivers and healthcare professionals in order to improve the safety of pharmacological treatment [37], a finding that is also supported by our study. In this context, community pharmacies can play a particularly relevant role as accessible sources of health information and support for caregivers.

Finally, it has been demonstrated that community pharmacists can make a significant contribution to optimizing medication use in frail older adults through activities such as health education, medication review, and the detection of medication-related problems [32].

### 3.1. Strengths and Limitations

Strengths

This study has several strengths. First, it included 148 community pharmacy professionals from urban, town, and rural settings, providing insight into caregiver-related medication errors across different geographical contexts. Although the rural subgroup was small, the inclusion of professionals from different pharmacy locations enabled the exploration of potential contextual variations in reported medication-safety practices. Second, the questionnaire addressed multiple dimensions of caregiver-related medication management, including the types of errors observed, contributing factors, preventive strategies, professional communication, perceived professional responsibilities and barriers affecting medication safety. This broad approach allowed medication errors to be examined not only as isolated events, but also in relation to caregiver support and community pharmacy practice.

Another strength is the inclusion of pharmacists, pharmacy technicians, and pharmacy assistants, which provides a broader perspective on how caregiver-related medication errors are identified and managed by different members of community pharmacy teams. By examining both the types of errors reported and the strategies used to detect, correct, and prevent them, the study offers a practice-oriented perspective that may help identify feasible opportunities for caregiver education and medication-safety interventions in routine community pharmacy practice.

Finally, the study addresses a relatively underexplored area by focusing specifically on caregiver-related medication errors from the perspective of community pharmacy professionals. This perspective may contribute to identifying practical opportunities for caregiver education, medication review, and early detection of medication-management difficulties in the home setting.

Limitations

The study is based on a self-administered questionnaire, which implies that the results reflect the subjective perceptions of the professionals rather than necessarily representing objective data on the actual incidence of medication errors. There is also the possibility that some responses may have been influenced by a tendency to provide socially desirable answers, particularly in questions related to professional attitude, empathy, or involvement in prevention. This limitation is particularly relevant because many respondents reported very high levels of professional involvement, empathy, communication skills, and willingness to intervene. These favourable responses may partly reflect social desirability bias; therefore, the findings should be interpreted as professionals’ reported perceptions and practices rather than as direct observations of behaviour.

Furthermore, although the sample size is relatively large, the distribution of responses to the questions addressed exclusively to professionals working in rural settings was limited, which may affect the stability of certain statistically significant results. The cross-sectional design also prevents causal interpretation of the observed associations. In addition, the small number of rural respondents (n = 11) may have produced low expected cell frequencies in some contingency tables. For this reason, findings involving rural pharmacies and statistically significant associations should be interpreted with caution. The large number of exploratory comparisons may also increase the probability of type I error, and no causal conclusions should be drawn from isolated significant results.

Moreover, the study does not directly assess the clinical consequences of the detected medication errors on patient health, which limits the extrapolation of the findings to specific clinical outcomes. Finally, because the study was based on professionals’ self-reported observations, it does not allow direct estimation of the actual frequency of medication errors in the population or the effectiveness of the preventive interventions described.

### 3.2. Clinical Implications

The findings of this study support the implementation of a core set of medication-safety strategies across community pharmacy settings, while allowing adaptation to local healthcare needs. Given that incorrect administration timing, omitted doses and dosing errors were the most frequently reported caregiver-related errors. Treatment complexity was the leading contributing factor, hence preventive interventions should prioritize a structured review of the complete medication regimen during dispensing encounters, particularly after treatment changes or transitions of care. This process may include confirming the current medication list, identifying discrepancies or potentially confusing instructions, assessing whether the caregiver understands the dose, administration schedule and route of administration. Medication reconciliation, pharmacist-led medication review, and pharmacotherapeutic follow-up may contribute to improve medication safety and optimizing treatment in older adults with polypharmacy [20,21,22].

These findings also support an expanded and proactive role for community pharmacists in medication safety at home. Beyond detecting and correcting errors during dispensing encounters, pharmacists may engage directly with informal caregivers by providing tailored education on medication doses, administration schedules, preparation techniques and the management of treatment changes. At the same time, they can actively verify, understand and identify practical difficulties before they result in medication errors. This educational role may be particularly relevant for preventing the recurrent error patterns identified in the present study, including incorrect administration timing, omitted doses, dosing errors and incorrect medication preparation.

Community pharmacists may also serve as an accessible source of medication-specific information for nursing professionals, thereby supporting nurses in their health-education activities aimed at preventing medication errors in the home setting [46]. Pharmacists can contribute detailed information on medication administration, dosage forms, interactions, adverse effects and treatment changes, while nurses may provide valuable information regarding the patient’s functional status, home-care environment and caregiver capacity [47]. This bidirectional exchange may promote more consistent education for patients and caregivers and strengthen interprofessional strategies for medication safety in the community.

When medication regimens are difficult for caregivers to manage, community pharmacy professionals may also identify opportunities for simplification; however, any modification affecting the prescribed treatment should be discussed and agreed with the prescriber. Caregiver education should be practical, individualized and focused on the tasks that must be performed at home. Verbal explanations in plain language may be complemented by updated written medication schedules, clear instructions following treatment changes, and active verification of understanding. The teach-back method may be particularly useful because it allows caregivers to explain, in their own words, how they intend to prepare and administer the medication, enabling misunderstandings to be identified before they result in an error [46]. Medication aids, including pill organizers or personalized dosage systems, may be useful when appropriately indicated, but they should complement rather than replace caregiver education, assessment, and follow-up.

Prevention of caregiver-related medication errors should be regarded as a shared responsibility involving pharmacists, physicians, nurses, patients, caregivers, and family members. Physicians retain responsibility for diagnosis and prescribing, whereas pharmacists may contribute by verifying the safe and appropriate use of medicines, detecting medication-related problems, providing counselling and communicating relevant concerns to the prescriber or other healthcare professionals. Nurses may also support medication education, monitoring, and continuity of care, particularly in patients with functional dependency or complex health needs. Caregivers should be recognized as active partners because they frequently organize, prepare, and administer medicines in the home and may be the first to identify practical difficulties or treatment-related problems [33,37]. Greater communication between all parties may help reduce fragmented information and improve continuity during treatment changes and transitions of care.

Although the findings suggest that similar core strategies may be applicable across urban, town, and rural settings, implementation should remain sensitive to local accessibility and caregiver-support needs. Rural-specific actions could include clearer referral pathways, coordinated follow-up and additional communication tools when access to other healthcare services is limited. Nevertheless, these proposals should be interpreted cautiously because the rural findings were based on only 11 respondents and were descriptive rather than comparative. Finally, the high levels of professional involvement and willingness to intervene reported in this study should be interpreted as evidence of favourable professional perceptions rather than as objective confirmation that community pharmacies are fully prepared to expand their responsibilities. Their implementation would require appropriate training, standardized protocols, sufficient resources, and effective interprofessional coordination.

## 4. Materials and Methods

### 4.1. Study Design

A cross-sectional questionnaire-based study was conducted using a self-reported online questionnaire to collect data on caregiver-related medication errors observed by community pharmacists, pharmacy technicians, and pharmacy assistants working in urban, town, and rural settings (see Supplementary Materials, Supplementary File S1).

The primary outcomes were the frequency and types of caregiver-related medication errors reported by community pharmacy professionals, as well as the frequency of specific preventive, corrective, and educational actions undertaken by pharmacists, pharmacy technicians, or pharmacy assistants according to pharmacy location.

In this study, caregivers were defined as individuals responsible for organizing medication for an older person, whether a family member or not. Medication error was defined as any prescribing error; omission error; incorrect administration time; administration of a non-prescribed medication; dosing error; administration error; or incorrect medication preparation.

The study was conducted in the Region of Murcia, Spain. Data collection from participating pharmacists, technicians, and assistants took place between November 2024 and January 2025. All participants agreed to take part with informed consent approved by the UCAM Ethics Committee under code CE022513.

### 4.2. Participants

The study sample consisted of pharmacists, technicians, and assistants working in community pharmacies located in urban areas, towns, or rural settings. Urban areas were defined as those with populations of more than 80.000 inhabitants, towns as those with populations between 3.000 and 80.000 and rural areas as those with populations between 100 and 3.000 inhabitants.

Inclusion criteria required participants to be 18 years of age or older, to commit to the study response deadlines, to be currently employed in a community pharmacy, and to hold the appropriate professional qualification for their position. All participants were salaried professionals and voluntarily agreed to complete the questionnaire based on their daily professional experience.

The medication errors collected in the study primarily referred to those committed by caregivers of older adults, while errors from other contexts were largely excluded. All identified errors were considered to have a direct impact on patient health. Participants also selected specific types of actions they routinely undertook when witnessing medication errors.

The required sample size was calculated to detect significant differences between caregiver-related medication errors, the actions performed by participants, and pharmacy location. The calculation assumed a 95% confidence interval and a ±20% margin of error.

Participants were recruited through voluntary participation following dissemination of the online questionnaire among community pharmacy professionals in the Region of Murcia. Therefore, a non-probability, convenience sampling approach was used. Recruitment continued until the target sample size was reached, resulting in a final sample of 148 participants, including pharmacists, pharmacy technicians, and pharmacy assistants.

### 4.3. Variables Studied

Data were collected using a self-administered questionnaire specifically designed for this study. The instrument aimed to evaluate pharmaceutical professionals’ actions in detecting and preventing medication errors committed by informal caregivers of older adults in the home setting, as well as the types of errors observed in daily practice.

The questionnaire included 40 questions addressed to all participants. Four of these consisted of multiple-choice practical case scenarios designed to assess the respondent’s ability to detect medication errors. Professionals working in rural settings answered six additional location-specific questions, resulting in a total of 46 questions. Of these 46, 12 were multiple-response questions allowing selection of all applicable options. Two of these were exclusively addressed to rural participants.

To organise the data analysis appropriately, the survey was structured into four sections. The first section included questions aimed at analysing the sociodemographic and professional characteristics of the respondents, such as age, sex, years of professional experience, professional category, and the setting in which they provided pharmaceutical care.

The second section included questions related to the medication errors most frequently identified by community pharmacy professionals in daily practice, including prescribing errors, omission errors, incorrect administration timing, administration of non-prescribed medication, dosing errors, administration errors, and incorrect medication preparation. For the purposes of this study, medication errors were defined as preventable events occurring during the prescribing, organization, dispensing, or administration of medicines that could compromise treatment safety or therapeutic effectiveness. This section also explored the main causes and contributing factors associated with these errors, such as treatment complexity, polypharmacy, communication and comprehension difficulties, limited caregiver training, caregiver burden, and barriers related to health literacy.

The third section focused on the strategies implemented by pharmacy professionals to detect, correct, and prevent medication errors, including medication review prior to dispensing, open-ended questioning, patient and caregiver counselling, and direct intervention when an error was identified.

Finally, the fourth section addressed variables related to professional communication with patients and caregivers, the perceived role of the pharmacist in medication safety and health education, the degree of professional involvement in error prevention, and the potential barriers associated with the geographical setting of the pharmacy, particularly in rural environments.

The questionnaire was made available through a link on an online platform, allowing participants to complete it without time restrictions. Respondents were required to answer all questions, leaving no items unanswered. Participation was voluntary and anonymous. Before accessing the questionnaire, participants were required to read the information about the study, including the definition of medication error, and provide informed consent.

### 4.4. Statistical Analysis

Prior to statistical analysis, the data were reviewed and organized into a structured database. Confidentiality was ensured, and all data were processed exclusively for academic and research purposes.

First, a descriptive analysis was conducted to characterize participants according to sociodemographic and professional variables. Categorical variables were summarized using absolute frequencies and percentages.

To examine potential associations between pharmacy location and questionnaire variables, contingency tables were constructed. For multiple-response questions and row-level comparisons, each response option was recoded as a binary variable indicating whether the option had been selected or not, allowing comparison of each category across urban, town, and rural pharmacies.

Categorical variables were summarised using absolute frequencies and percentages. Associations between pharmacy location and categorical variables were assessed using Pearson’s chi-square (χ^2^) test when its assumptions were met. Expected cell frequencies were examined for each comparison. When the assumptions of Pearson’s chi-square test were not satisfied because of low expected cell counts, *p*-values were calculated using the Fisher–Freeman–Halton exact test for 2 × 3 contingency tables. Statistical significance was set at *p* < 0.05, and all tests were two-sided. Eta squared (η^2^), calculated as χ^2^/N, was retained as a descriptive chi-square-based index of the magnitude of the observed associations. Given the small number of respondents in the rural subgroup and the exploratory nature of the multiple comparisons, statistically significant findings were interpreted cautiously. To complement statistical significance testing, effect size was estimated using eta squared (η^2^) as a chi-square-based descriptive index calculated as χ^2^/N. Although eta squared is most used in analyses involving differences in means, such as ANOVA, in the present study it was retained as a descriptive index of the magnitude of association derived from the chi-square statistic. These values should therefore be interpreted cautiously as descriptive indicators of association rather than as measures of explained variance or causality. The level of statistical significance was set at *p* < 0.05. Given the exploratory nature of the study and the number of comparisons performed, statistically significant findings were interpreted cautiously. All analyses were performed using SPSS version 29.0 (IBM Corp, Armonk, NY, USA).

## 5. Conclusions

In conclusion, this study suggests that community pharmacy professionals report strong engagement in medication error detection and prevention across settings. Differences were mainly related to perceived error frequency and expanded rural roles. In the context of population ageing and increasing therapeutic complexity, community pharmacies should be considered one important component of a wider patient-safety strategy, together with prescribers, caregivers, relatives, and other healthcare professionals.

## Figures and Tables

**Table 1:** Professionals’ demographic description.

Variable	City n = 74 (50%)	Town n = 63 (43%)	Rural n = 11 (7%)	*p*-Value	η^2^
**Age 20–39 years**	25 (33.8%)	21 (33.3%)	3 (27.3%)	1.0000	0.0013
**Age 40–59 years**	38 (51.4%)	33 (52.4%)	2 (18.2%)	0.0990	0.0313
**Age 60–80 years**	11 (14.9%)	9 (14.3%)	6 (54.5%)	0.0099	0.0759
**Male**	16 (21.6%)	23 (36.5%)	8 (72.7%)	0.0020	0.0857
**Female**	58 (78.4%)	40 (63.5%)	3 (27.3%)	0.0020	0.0857
**Experience < 5 years**	16 (21.6%)	14 (22.2%)	2 (18.2%)	1.0000	0.0006
**Experience 5–15 years**	17 (23.0%)	11 (17.5%)	3 (27.3%)	0.5729	0.0062
**Experience 16–25 years**	18 (24.3%)	17 (27.0%)	0 (0.0%)	0.1397	0.0258
**Experience 26–30 years**	12 (16.2%)	13 (20.6%)	1 (9.1%)	0.7154	0.0071
**Experience > 30 years**	11 (14.9%)	8 (12.7%)	5 (45.5%)	0.0355	0.0513
**Pharmacist**	57 (77.0%)	43 (68.3%)	9 (81.8%)	0.4984	0.0119
**Technician**	12 (16.2%)	12 (19.0%)	0 (0.0%)	0.3534	0.0169
**Assistant**	5 (6.8%)	8 (12.7%)	2 (18.2%)	0.2273	0.0146
**Counter service**	70 (94.6%)	56 (88.9%)	10 (90.9%)	0.4482	0.0102
**Consultation room service**	3 (4.1%)	7 (11.1%)	1 (9.1%)	0.1978	0.0170
**Telephone/WhatsApp service**	1 (1.4%)	0 (0.0%)	0 (0.0%)	1.0000	0.0068

Note: *p*-values were obtained using Pearson’s chi-square test when assumptions were met and Fisher–Freeman–Halton exact tests when expected cell counts were below the recommended threshold. η^2^ was retained as a chi-square-based descriptive index (χ^2^/N).

**Table 2:** Medication errors and associated factors.

	Option	City n = 74 (50%)	Town n = 63 (43%)	Rural n = 11 (7%)	*p*	η^2^
**Error Types**	Prescribing error	12 (16.2%)	12 (19.0%)	0 (-)	0.3475	0.0169
	Omission error	45 (60.8%)	36 (57.1%)	6 (54.5%)	0.8468	0.0019
	Administration time	47 (63.5%)	37 (58.7%)	7 (63.6%)	0.8693	0.0024
	Non-prescribed meds	26 (35.1%)	20 (31.7%)	4 (36.4%)	0.8585	0.0014
	Dosing error	44 (59.5%)	31 (49.2%)	5 (45.5%)	0.4077	0.0121
	Different dosage form	6 (8.1%)	5 (7.9%)	0 (-)	1.0000	0.0065
	Incorrect preparation	32 (43.2%)	16 (25.4%)	1 (9.1%)	0.0190	0.0540
	Administration error	33 (44.6%)	24 (38.1%)	4 (36.4%)	0.7547	0.0048
**Error Causes**	Lack of health literacy	47 (63.5%)	34 (54.0%)	6 (54.5%)	0.4722	0.0092
	Treatment complexity	65 (87.8%)	49 (77.8%)	10 (90.9%)	0.2698	0.0201
	Caregiver distractions	34 (45.9%)	27 (42.9%)	6 (54.5%)	0.7378	0.0037
	Communication issues	50 (67.6%)	50 (79.4%)	8 (72.7%)	0.2808	0.0162
**Error Factors**	Lack of training	46 (62.2%)	44 (69.8%)	6 (54.5%)	0.4598	0.0097
	Complex treatments	55 (74.3%)	39 (61.9%)	8 (72.7%)	0.3143	0.0171
	Caregiver burden	38 (51.4%)	28 (44.4%)	6 (54.5%)	0.6652	0.0055
	Poor communication	45 (60.8%)	40 (63.5%)	7 (63.6%)	0.9654	0.0008
**Errors in Forms**	Solid	45 (60.8%)	29 (46.0%)	4 (36.4%)	0.1192	0.0287
	Liquid	7 (9.5%)	6 (9.5%)	1 (9.1%)	1.0000	0.0000
	Gaseous	22 (29.7%)	28 (44.4%)	6 (54.5%)	0.0992	0.0307
**Errors in Routes**	Oral	48 (64.9%)	36 (57.1%)	7 (63.6%)	0.6278	0.0059
	Topical	0 (-)	0 (-)	0 (-)	NA	NA
	Parenteral	0 (-)	0 (-)	0 (-)	NA	NA
	Rectal	0 (-)	0 (-)	0 (-)	NA	NA
	Vaginal	0 (-)	0 (-)	0 (-)	NA	NA
	Inhalation	24 (32.4%)	26 (41.3%)	4 (36.4%)	0.5609	0.0077
	Ophthalmic	2 (2.7%)	1 (1.6%)	0 (-)	1.0000	0.0031
	0 (-)	0 (-)	0 (-)	0 (-)	NA	NA
**Errors by Groups**	Antihypertensives	37 (50.0%)	24 (38.1%)	4 (36.4%)	0.3263	0.0151
	Hypoglycaemic agents	31 (41.9%)	29 (46.0%)	8 (72.7%)	0.1599	0.0248
	Anti-inflammatories	45 (60.8%)	32 (50.8%)	5 (45.5%)	0.3810	0.0126
	Analgesics	45 (60.8%)	26 (41.3%)	6 (54.5%)	0.0729	0.0354

Note: *p*-values were obtained using Pearson’s chi-square test when assumptions were met and Fisher–Freeman–Halton exact tests when expected cell counts were below the recommended threshold. η^2^ was retained as a chi-square-based descriptive index (χ^2^/N). NA, not applicable because the test could not be computed due to absence of variability.

**Table 3:** Error detection and prevention strategies.

	Response Option	City n = 74 (50%)	Town n = 63 (43%)	Rural n = 11 (7%)	*p*	η^2^
**Error detection**	Open-ended questions about medications intake	67 (90.5%)	51 (81.0%)	11 (100.0%)	0.1213	0.0307
	Medication conciliation	42 (56.8%)	40 (63.5%)	8 (72.7%)	0.5516	0.0092
	Observing confusion	50 (67.6%)	34 (54.0%)	5 (45.5%)	0.1563	0.0250
	Pharmacotherapeutic follow-up	14 (18.9%)	8 (12.7%)	2 (18.2%)	0.6125	0.0068
**Complex Conditions requiring explanations or advice to avoid errors**	Hypertension and cardiovascular diseases	45 (60.8%)	36 (57.1%)	7 (63.6%)	0.8689	0.0019
	Diabetes	50 (67.6%)	34 (54.0%)	11 (100.0%)	0.0068	0.0633
	Neurological or psychiatric diseases	57 (77.0%)	35 (55.6%)	8 (72.7%)	0.0275	0.0493
	Chronic pain	31 (41.9%)	22 (34.9%)	2 (18.2%)	0.2995	0.0172
	Infectious diseases	33 (44.6%)	21 (33.3%)	1 (9.1%)	0.0519	0.0396
	Respiratory conditions	44 (59.5%)	44 (69.8%)	5 (45.5%)	0.2185	0.0210
	Ophthalmic conditions	18 (24.3%)	13 (20.6%)	2 (18.2%)	0.9078	0.0026
	Other	3 (4.1%)	0 (-)	0 (-)	0.4112	0.0207
**Ways to ensure that the caregiver has understood the information correctly**	Information repetition	58 (78.4%)	45 (71.4%)	11 (100.0%)	0.0984	0.0302
	Providing Written information	51 (68.9%)	35 (55.6%)	8 (72.7%)	0.2269	0.0207
	Asking specific questions	39 (52.7%)	27 (42.9%)	5 (45.5%)	0.5087	0.0091
	Medication follow-up via WhatsApp or phone call	2 (2.7%)	4 (6.3%)	1 (9.1%)	0.2934	0.0102
**Medication aids recommendations to help caregiver with medication**	Use of pill organisers	57 (77.0%)	43 (68.3%)	8 (72.7%)	0.5169	0.0090
	Offer of the PDS service	55 (74.3%)	43 (68.3%)	6 (54.5%)	0.3778	0.0136
	Use of alarms or reminders	18 (24.3%)	13 (20.6%)	3 (27.3%)	0.7926	0.0026
	Placement of notes in highly visible places	8 (10.8%)	7 (11.1%)	1 (9.1%)	1.0000	0.0003
	Written delivery of all information	38 (51.4%)	28 (44.4%)	7 (63.6%)	0.4441	0.0110
**Strategies for follow-up and prevention of caregiver errors**	Periodic review of the medication at each dispensing	58 (78.4%)	45 (71.4%)	9 (81.8%)	0.6650	0.0077
	Scheduled follow-up appointments	2 (2.7%)	6 (9.5%)	0 (-)	0.2321	0.0255
	Communication with provider	4 (5.4%)	1 (1.6%)	0 (-)	0.5804	0.0131
	Continuous education	57 (77.0%)	48 (76.2%)	11 (100.0%)	0.2156	0.0222
**Frequency of intervention in prescribing errors**	Whenever I detect them	70 (94.6%)	59 (93.7%)	11 (100.0%)	1.0000	0.0050
	Only if it is serious	3 (4.1%)	0 (-)	0 (-)	0.4094	0.0207
	Sometimes, if I have time	1 (1.4%)	2 (3.2%)	0 (-)	0.6825	0.0055
	Rarely, it is not my function	0 (-)	2 (3.2%)	0 (-)	0.3220	0.0185
**Strategies to educate about medication errors and prevent them**	Explain with plain language	66 (89.2%)	58 (92.1%)	10 (90.9%)	0.9045	0.0022
	Provide a leaflet with recommendation	3 (4.1%)	3 (4.8%)	0 (-)	1.0000	0.0037
	Refer to the physician	1 (1.4%)	0 (-)	1 (9.1%)	0.1449	0.0392
	Not usually do it	4 (5.4%)	2 (3.2%)	0 (-)	0.8112	0.0063

Note: *p*-values were obtained using Pearson’s chi-square test when assumptions were met and Fisher–Freeman–Halton exact tests when expected cell counts were below the recommended threshold. η^2^ was retained as a chi-square-based descriptive index (χ^2^/N).

**Table 4:** Professional attitudes and communication.

	Response Option	City n = 74 (50%)	Town n = 63 (43%)	Rural n = 11 (7%)	*p*	η^2^
**Ability to communicate medication risks to caregivers**	Very good	17 (23.0%)	19 (30.2%)	2 (18.2%)	0.6132	0.0086
	Good	48 (64.9%)	30 (47.6%)	7 (63.6%)	0.1150	0.0292
	Needs improvement	9 (12.2%)	14 (22.2%)	2 (18.2%)	0.2657	0.0167
	Insufficient	0 (-)	0 (-)	0 (-)	NA	NA
**Pharmacist reaction to a medication error by the caregiver**	I delegate error management	1 (1.4%)	2 (3.2%)	0 (-)	0.6744	0.0055
	I listen to the caregiver and re-schedule them	7 (9.5%)	2 (3.2%)	0 (-)	0.2752	0.0211
	I feel anxious and do not know how to address it	0 (0.0%)	2 (3.2%)	0 (-)	0.3233	0.0185
	I correct caregiver immediately	66 (89.2%)	57 (90.5%)	11 (100.0%)	0.7575	0.0088
**Pharmacy involvement in preventing medication errors**	I get involved as a part of our professional role	41 (55.4%)	34 (54.0%)	8 (72.7%)	0.5833	0.0092
	As far as the patient allows	32 (43.2%)	26 (41.3%)	3 (27.3%)	0.6707	0.0068
	If there is a complaint or a clinical consequence	1 (1.4%)	1 (1.6%)	0 (-)	1.0000	0.0012
	It is the responsibility of the physician and the family	0 (0.0%)	2 (3.2%)	0 (-)	0.3260	0.0185
**Empathy towards people with health impairments**	High	58 (78.4%)	54 (85.7%)	10 (90.9%)	0.4697	0.0125
	Moderate	16 (21.6%)	9 (14.3%)	1 (9.1%)	0.4608	0.0125
	Low	0 (-)	0 (-)	0 (-)	NA	NA
	None	0 (-)	0 (-)	0 (-)	NA	NA
**Relationship between communication style and patient adherence**	A great deal, I take great care	64 (86.5%)	56 (88.9%)	11 (100.0%)	0.5781	0.0117
	It depends on the day	8 (10.8%)	7 (11.1%)	0 (-)	0.7666	0.0091
	Technical information is what matters	1 (1.4%)	0 (-)	0 (-)	1.0000	0.0068
	It has no influence	1 (1.4%)	0 (-)	0 (-)	1.0000	0.0068
**Level of preparedness to correct medication errors without generating guilt**	Very prepared	22 (29.7%)	16 (25.4%)	3 (27.3%)	0.9218	0.0022
	Prepared	49 (66.2%)	43 (68.3%)	8 (72.7%)	0.9657	0.0014
	Little prepared	3 (4.1%)	4 (6.3%)	0 (-)	0.8293	0.0067
	Not prepared at all	0 (-)	0 (-)	0 (-)	0.6132	0.0086

Note: *p*-values were obtained using Pearson’s chi-square test when assumptions were met and Fisher–Freeman–Halton exact tests when expected cell counts were below the recommended threshold. η^2^ was retained as a chi-square-based descriptive index (χ^2^/N). NA, not applicable because the test could not be computed due to absence of variability.

**Table 5:** Rural pharmacists’ perspectives.

Question	Answer	n = 11 (7.4%)
**Carrying out additional educational functions that are not so common**	Frequently	2 (18.2%)
	Occasionally	6 (54.5%)
	Sporadically	3 (27.3%)
**Feeling of an educational role and duty to provide emotional support to caregivers**	Frequently	2 (18.2%)
	Sometimes	7 (63.6%)
	Never	2 (18.2%)
**Frequency of errors due to geographical barriers**	A lot	7 (63.6%)
	Quite a lot	1 (9.1%)
	A little	3 (27.3%)
**Types of barriers in the rural setting**	Low health literacy	3 (27.3%)
	Limited digital access	2 (18.2%)
	Caregiver loneliness	3 (27.3%)
	Lack of health training	7 (63.6%)
	Distance to the health centre	3 (27.3%)
	Lack of family coordination	5 (45.5%)

Note: Table 5 is descriptive only because it includes rural respondents exclusively; therefore, inferential comparisons between pharmacy locations were not performed.

## Data Availability

The datasets generated and/or analyzed during the current study are available from the corresponding author on reasonable request.

## Supplementary Materials

The following supporting information can be downloaded at: https://www.mdpi.com/article/10.3390/ph19081148/s1, Supplementary File S1: Rafael Toledo Ruiz—Questionnaire: Medication Errors by Caregivers of Elderly People.
