# Supplementary material for: Observation and Prevention of Caregiver-Related Medication Errors in Community Pharmacies: A Cross-Sectional Study Across Urban, Town, and Rural Settings
Source: Pharmaceuticals (Basel). 2026 Jul 24;19(8):1148. doi: 10.3390/ph19081148 (PMC13516728; doi:10.3390/ph19081148)
Supplement: Supplementary file 1 [file pharmaceuticals-19-01148-s001.zip › pharmaceuticals-4372271-supplementary.pdf]

RAFAEL TOLEDO RUIZ

QUESTIONNAIRE

MEDICATION ERRORS BY CAREGIVERS OF ELDERLY PEOPLE

In this questionnaire, *caregivers* are understood to be those individuals who organize the medication of an elderly person (whether a family member or not). A son who prepares his mother's medication is considered a caregiver (it is not necessary for them to work officially as a paid caregiver).

Main medication errors:

- Prescription error: the doctor has prescribed a medication different from the one the patient needed.
- Omission error: the patient forgets to take a dose, for example.
- Incorrect administration time.
- Administration of a non-prescribed medication.
- Dosage error: the dose is incorrect.
- Medication preparation error: incorrect dilution, reconstitution, or mixing of medications that are physically or chemically incompatible.
- Administration error: incorrect route of administration or incorrect conditions of administration (with or without food, with other medications, etc.).

Informed consent

I have read and accept the terms stated in the informed consent

Personal information

1. Full name

2. Age (answer with a number)

3. Gender:

☐ Female

☐ Male

4. Email address

5. Years of experience in a community pharmacy (answer with a number)

6. What is your role in the pharmacy?

☐ Pharmacist

☐ Technician

☐ Assistant

7. Where do you provide pharmaceutical care? (multiple choice)

☐ Counter

☐ Consultation room

☐ Telephone

☐ WhatsApp

8. What medication errors do you observe? (multiple choice)

☐ Prescribing error

☐ Omission error

☐ Wrong administration time

☐ Non-prescribed medication

☐ Dosage error

☐ Wrong pharmaceutical form

☐ Incorrect preparation

☐ Administration error

9. Causes of errors (multiple choice)

☐ Lack of knowledge

☐ Treatment complexity

☐ Caregiver overload

☐ Communication problems

10. Factors increasing errors (multiple choice)

☐ Age >65

☐ Number of medications

☐ Living situation

☐ Dependency

☐ Education

☐ Emotional burden

11. Detection methods (multiple choice)

☐ Open questions

☐ Medication review

☐ Observation

☐ Follow-up

12. Pharmaceutical form most associated with errors

☐ Solid

☐ Semi-solid

☐ Liquid

☐ Gaseous

13. Route of administration most associated with errors

☐ Oral

☐ Topical

☐ Parenteral

☐ Rectal

☐ Vaginal

☐ Inhalation

☐ Ophthalmic

☐ Otic

14. Medication groups prone to errors (multiple choice)

☐ Analgesics

☐ Anti-inflammatory

☐ Antihistamines

☐ Anti-infectives

☐ Anesthetics

☐ Diuretics

☐ Antihypertensives

☐ Hypoglycemics

☐ Antiemetics

☐ Antidiarrheals

☐ Others

15. Influence of packaging design

☐ Not at all

☐ A little

☐ Quite a lot

☐ A lot

16. Influence of pill color

☐ Not at all

☐ A little

☐ Quite a lot

☐ A lot

17. Conditions requiring more explanation (multiple choice)

☐ cardiovascular diseases and hypertension

☐ Diabetes

☐ Neurological/psychiatric

☐ Chronic pain

☐ Infectious diseases

☐ Respiratory diseases

☐ Ophthalmic diseases

☐ Others

18. How do you ensure understanding? (multiple choice)

☐ Repeat information

☐ Written info

☐ Ask questions

☐ Follow-up contact (WhatsApp or phone call)

19. Advice to prevent missed doses (multiple choice)

☐ Pill organizers

☐ SPD system

☐ Alarms

☐ Sticky notes

☐ Written instructions

20. Have you observed that medication errors among caregivers of older adults vary according to any of the following factors? (multiple choice)

☐ Low education

☐ Advanced age

☐ Emotional state

21. What strategies do you use to carry out follow-up and prevent medication errors among caregivers? (multiple choice)

☐ Regular medication review at each dispensing

☐ Scheduled follow-up appointments

☐ Ongoing communication with the physician

☐ Continuous education of the caregiver or older patient at each pharmacy visit

22. Do you encounter overwhelmed caregivers?

☐ Frequently

☐ Occasionally

☐ Rarely

☐ Never

23. How often do you actively intervene to correct prescribing errors?

☐ Always

☐ Only if serious

☐ Sometimes

☐ Rarely

24. How do you rate your ability to communicate medication-related risks to caregivers?

☐ Very good

☐ Good

☐ Needs improvement

☐ Poor

25. How do you react when you detect a medication error made by a caregiver?

☐ I delegate the management of the error to another colleague

☐ I calmly listen to the caregiver and ask them to come back another day

☐ I feel anxious and am not sure how to handle the situation

☐ I correct them immediately in a clear and direct manner

26. How often do you detect medication errors made by caregivers of older adults?
☐ Frequently
☐ Occasionally
☐ Rarely
☐ I do not usually pay attention to this type of issue

27. How often do you detect prescribing errors made by physicians?
☐ Frequently
☐ Occasionally
☐ Rarely
☐ Never

28. What do you think when you see that a caregiver has made the same error several times?
☐ They are unmotivated in their job
☐ They do not care about the errors they make; they do not consider it part of their role
☐ They need more support or education
☐ They do not fully understand the error they have made

29. How often do caregivers make the same error?
☐ Frequently
☐ Occasionally
☐ Rarely
☐ Never

30. To what extent do you think the pharmacy should be involved in preventing medication errors made by caregivers of older adults?
☐ It should be fully involved; it is part of our professional role regardless of the patient's opinion
☐ To the extent that the patient allows it
☐ Only if there is a complaint or a possible clinical consequence
☐ Very little; it is the responsibility of the physician and the family

31. What level of empathy do you think you have towards older adults with cognitive or functional difficulties?

☐ High

☐ Moderate

☐ Low

☐ None

32. What do you say to the caregiver when you realize they have made an error?

☐ I explain it kindly, without reproach, and in a concise way

☐ I explain it in a more technical and rigorous way and occasionally reproach them

☐ I hold them responsible for the error

☐ I agree with their objections as I prefer not to confront them

33. What do you do if the caregiver minimizes the error made?

☐ I try to explain the consequences in clear and simple language

☐ I provide written informational material

☐ If the error is not very serious, I do not say anything

☐ I exaggerate the error to scare them so they do not repeat it

34. How do you think your way of communicating with the caregiver influences patient adherence?

☐ Very much; I am very careful with how I express myself

☐ Somewhat; it depends on the day

☐ Little; the important thing is the technical information

☐ Not at all; correct medication use is solely the patient’s responsibility

35. How often do you adapt your way of speaking to the cognitive and educational level of the caregiver or older patient?

☐ Always

☐ Most of the time

☐ Sometimes I forget

☐ Rarely; I do not know how to do it

36. How do you manage your time to properly address medication errors made by caregivers?

☐ I take the necessary time even if there is a queue

☐ I look for quiet moments

☐ I find it difficult to find the right moment

☐ I tend to avoid them due to lack of time

37. Do you use any strategies to educate about medication errors and prevent them?

☐ I explain using simple examples and repeat if necessary

☐ I provide a leaflet and give a recommendation

☐ I refer to the physician

☐ I do not usually do this

38. Do you think the pharmacy should have a more active role in pharmacological follow-up?

☐ Yes, completely

☐ Only in high-risk patients

☐ No, that exceeds our responsibilities

☐ It depends on the patient

39. What tools would you need to more effectively address and prevent medication errors?

☐ Training in communication with older adults and caregivers

☐ Specific protocols and intervention tools

☐ More time at the counter

☐ Psychological support or group sessions with colleagues

40. How often do you actively ask caregivers if they understand or have any questions about the treatment?

☐ Always

☐ Almost always

☐ From time to time

☐ Never

41. How prepared do you feel to correct medication errors in caregivers without making them feel guilty or ashamed?

☐ Very prepared

☐ Prepared

☐ Slightly prepared

☐ Not prepared at all

42. How do you rate your ability to identify medication errors when the caregiver has a low educational level or difficulty understanding written instructions?

☐ Very high

☐ High

☐ Moderate

☐ Low

HYPOTHETICAL REAL CASES

Case 1

A 57-year-old caregiver comes to the pharmacy and, while speaking with you spontaneously, mentions that their mother does not remember whether she took her cholesterol pill yesterday. The caregiver told her this morning to take two pills together just in case, to make up for the missed dose.

What type of error do you observe?

- Prescribing error
- Omission error
- Wrong administration time
- Administration of a non-prescribed medication
- Dosage error
- Incorrect preparation of the medication (wrong dilution, reconstitution, or incompatible mixtures)
- Administration error (wrong route or conditions of administration)

Case 2

An 82-year-old man with hypertension says he has shoulder pain and took a 650 mg paracetamol tablet, but it "did not work." The caregiver told him there was no need to see the doctor and suggested he try 600 mg ibuprofen that was left over from a previous treatment.

What type of error do you observe?

- Prescribing error
- Omission error
- Wrong administration time
- Administration of a non-prescribed medication
- Dosage error
- Administration of a different pharmaceutical form than prescribed
- Incorrect preparation of the medication
- Administration error

Case 3

A caregiver repeatedly asks the same question every time they come to the pharmacy because they cannot remember when the older person should take Sintrom. They say they alternate the timing, taking it one day with lunch and the next day with dinner.

What type of error do you observe?

- Prescribing error
- Omission error
- Wrong administration time
- Administration of a non-prescribed medication
- Dosage error
- Administration of a different pharmaceutical form than prescribed
- Incorrect preparation of the medication
- Administration error

Case 4

A caregiver splits a prolonged-release capsule to make it easier for their father, who has dysphagia, to take it.

What type of error do you observe?

- Prescribing error
- Omission error
- Wrong administration time
- Administration of a non-prescribed medication
- Dosage error (higher or lower than prescribed)
- Administration of a different pharmaceutical form than prescribed
- Incorrect preparation of the medication
- Administration error

47. What type of pharmacy do you work in?

☐ Urban: high-traffic area

☐ Urban: residential area/neighborhood

☐ Urban: area with predominantly older population

☐ Town: main street

☐ Town: neighborhood

☐ Rural area (very small or sparsely populated areas)

Answer the following questions related to the rural setting

Have you had to take on educational or follow-up roles that are less common in an urban pharmacy in order to prevent medication errors made by caregivers of older adults?

☐ Frequently

☐ From time to time

☐ Occasionally, but rarely

☐ Never

How often do you detect medication errors made by caregivers due to geographical barriers or lack of access to healthcare professionals?

☐ Always

☐ Frequently

☐ Sometimes

☐ Never

To what extent do you feel that your role as a rural pharmacist includes educating and providing emotional support to caregivers of older adults?

☐ A lot

☐ Quite a lot

☐ A little

☐ Not at all

How often have you had to change your way of working or adapt your interventions due to the absence or distance of the physician?

☐ Always

☐ Frequently

☐ Sometimes

☐ Never

What specific barriers (e.g., low health literacy, limited digital access, caregiver loneliness) have you identified in your rural setting that make proper medication management more difficult for caregivers?
(You may select more than one option)

☐ Low level of health literacy

☐ Limited access to digital resources

☐ Caregiver loneliness

☐ Lack of healthcare training

☐ Distance and/or difficulty accessing a hospital or healthcare center

☐ Lack of coordination among family caregivers

If you could have an additional resource in your rural practice to help prevent medication errors made by caregivers, what would it be (training, technology, coordination, psychological support, etc.) and why?

☐ Training

☐Technological tools

☐ Visual or written materials

☐ Coordination among healthcare professionals

☐ Psychological support
